# Synthesis, structural and electrochemical properties of V_4_O_9_ cathode for lithium batteries

**DOI:** 10.3389/fchem.2023.1161053

**Published:** 2023-04-20

**Authors:** Premkumar Senguttuvan, Eungje Lee, Baris Key, Christopher S. Johnson

**Affiliations:** ^1^ Chemical Sciences and Engineering Division, Lemont, IL, United States; ^2^ Argonne National Laboratory, Joint Center Energy Storage Research, Lemont, IL, United States

**Keywords:** vanadium oxide, lithium, batteries, layered, cathode, V_2_O_5_, V_4_O_9_

## Abstract

Single-phase three-dimensional vanadium oxide (V_4_O_9_) was synthesized by reduction of V_2_O_5_ using a gas stream of ammonia/argon (NH_3_/Ar). The as-synthesized oxide, prepared by this simple gas reduction method was subsequently electrochemically transformed into a disordered rock salt type-“Li3.7V4O9” phase while cycling over the voltage window 3.5 to 1.8 V versus Li. The Li-deficient phase delivers an initial reversible capacity of ∼260 mAhg^−1^ at an average voltage of 2.5 V vs. Li^+^/Li^0^. Further cycling to 50 cycles yields a steady 225 mAhg^−1^. *Ex situ* X-ray diffraction studies confirmed that (de) intercalation phenomena follows a solid-solution electrochemical reaction mechanism. As demonstrated, the reversibility and capacity utilization of this V_4_O_9_ is found to be superior to battery grade, micron-sized V_2_O_5_ cathodes in lithium cells.

## Highlights

• Low temperature ammonia gas chemical reduction of V_2_O_5_ creates V_4_O_9_, a newly formed corner shared polyhedral VO_x_ structure
• V_4_O_9_ demonstrates reversible smooth lithium (de) intercalation profiles
• Capacities as high as 225 mAhg^−1^ are demonstrated between 3.5 to 1.8 V vs. Li metal

## Introduction

Classic electrochemically active crystalline vanadium oxides such as V_2_O_5_ and the lithium-containing version, LiV_3_O_8_, have long been evaluated in lithium batteries as cathodes owing to their high theoretical capacities when cycled to rock salt type composition; i.e., Li_3_V_2_O_5_ and Li_5_V_3_O_8_ (Panero et al., 1983; Delmas et al., 1991; Delmas et al., 1994; West et al., 1995; Kawakita et al., 1998; Jouanneau et al., 2005; Leger et al., 2005; Chernova et al., 2009). For example, the theoretical lithium (de) intercalation capacities for V_2_O_5_ is 440 mAhg^−1^, and for LiV_3_O_8_ is 372 mAhg^−1^. Thus, notable high battery energy densities can result for full cells when coupled with metallic Li anodes above ca. 481 Wh kg^−1^. These high energy densities are linked to the electrochemical activity origin associated with multiple V(V) to V(III) redox state changes and ready insertion of lithium cations (Cartier et al., 1990). However, phase transitions transpire between multiple oxidation states that drive structural changes in these materials which can lead to cycling fade (Delmas et al., 1994). On the other hand, another layered phase LiVO_3_ has exhibited good capacity retention after the transformation into rock salt phase due to restricted electrochemical activity to V^5+^/V^4+^ redox couple but with lower capacity (Pralong et al., 2012).

The early work of S. Passerini et al. (Appetecchi et al., 2001) has focused on V_2_O_5_ cathodes versus Li foil coupled with a thin polymer electrolyte membrane/separator, such as polyethylene oxide (PEO) plasticized with LiTFSI salt (large amorphous polymer region). Perhaps the earliest successful work that employed vanadium oxide-type materials was the use of LiV_3_O_8_ with both liquid ester carbonate solvents and PEO polymer electrolyte systems (Bonino et al., 1988). Recently nanotechnology enhancements have taken place, and nanoflowers of V_2_O_5_ have also been reported with extremely high capacities of 275 mAh/g after 50 cycles (liquid ester carbonate electrolyte based) (Tang et al., 2013). Other nanosized materials such as nanobelts (Li et al., 2006), and nanowires (Li et al., 2007) of V_2_O_5_ tested in lithium batteries have been investigated, but these materials are complicated to synthesize and the volumetric energy density is compromised by the nanometer dimensionality and requisite low electrode loadings (Wang and Cao, 2006). Micron-sized hollow spheres of V_2_O_5_ were also reported (Zhu et al., 2008), thus demonstrating that vanadium oxides can reach an effective capacity of 319 mAh/g between 4.0 and 2.0 V cutoffs. After 50 cycles, however, the capacity diminishes to about 210–220 mAh/g in Li cell. Lastly, VO_2_ (B) phase is electrochemically active and has been studied as a novel cathode which converts to a Zn-VO_x_ material in a aqueous Zn battery (Ding et al., 2021).

In the context of Li metal based liquid electrolyte cells, the promise of 500 Wh/kg pouch-format cells is within reach with high Ni cathodes. (Liu et al., 2019). Certainly Li metal allows such an extreme energy dense system, but also brings in the opportunity to use charged (non-lithium containing) electrodes like V_2_O_5_ or other vanadium oxide phases. An exploitation of the high capacity from two electrons from V(V) to V(III) in such systems may be possible. The Li cells are discharged first thus inserting lithium cations into the host vanadium oxide. Thus far, however, many issues of constricted Li-ion motion, vanadium atomic movement and resultant coordination distortion causes phase changes in the host material (De Jesus et al., 2018), which adversely effects stability and lowering of capacity. In this present work we focus on the synthesis of a different structure; V_4_O_9_ with large one-dimensional tunnels, and present the electrochemical cycling, and associated mechanism of structural change for the first time. Cycling leads to a more stable crystalline lithiated rock-salt phase with good reversibility and high capacities in Li cells. We compare the capacity retention and values to battery-grade micron-sized polycrystalline V_2_O_5_ electrode and lend evidence to why the cycling is superior. We find that the V_4_O_9_ follows a different reaction pathway than V_2_O_5_, resulting in a more stable structure amiable to reversible and smooth (de) intercalation processes.

## Experimental

Synthesis of V_4_O_9_ was carried out by reducing V_2_O_5_ precursor under a flow of 3 mol% ammonia (NH_3_) in argon (Ar) gas stream (AGA gas; 99.9%). The ammonia/Ar gas flow was 20 cc/min. A gas outlet trap from the tube furnace was filled with dilute acetic acid to neutralize the effluent ammonia gas. Typically 500 mg of V_2_O_5_ (Sigma, 99.6%) was placed in an alumina crucible inside a tubular furnace and heat treatment was carried out at different temperatures for 12 h. Vanadium oxide (V_2_O_5_), was purchased (Sigma-Aldrich 99%) and used without further purification. It also was ground and sieved prior to electrode making and electrochemical testing. The purity grade for the electrochemical sample was slightly lower, 99%, versus 99.6% for the synthesis precursor sample. The impact on capacity value is expected to be less than 1%, as the impurities are less than 1%.

Structural characterization was carried out using a Rigaku lab diffractometer (Cu Kα radiation) at a scan rate of 2.0°/min in between 10° and 60° 2θ range. Additional synchrotron-based powder SXRD patterns were collected from 11-BM (Sector 11 bending magnet line) at the Argonne National Laboratory’s Advanced Photon Source. The SXRD patterns were fitted and indexed using Le Bail whole-powder pattern decomposition method.

Electrochemical tests were carried out in coin cell configuration (2032 size; Hohsen) assembled in an argon-filled glovebox (H_2_O and O_2_ < 0.1 ppm). Cathodes were prepared from a slurry mixture (solvent: N-methylpyrrolidinone; Sigma-Aldrich, 99 + %) of 80wt% V_4_O_9_ powder, 12wt% carbon black (Super P-Li, Cabot Co.,) and 8wt% poly (vinylidene fluoride) (PVDF, Kynar) which was then coated on an aluminum foil current collector. The electrode laminate was dried at 70°C in a vacuum oven, calendared and punched out as a disc (9/16 in. diameter) and contained an active mass of 3 mg cm^−2^. The anode was a lithium metal disc (Hohsen), and the electrolyte (Tomiyama chemical) consisted of 1.2 M LiPF_6_ in a mixture of ethylene carbonate and ethyl methyl carbonate (3:7 by weight). These coin cells were set to cycle between 1.8 and 3.5 V vs. Li^+^/Li^0^ at C/10 rate. Likewise the V_2_O_5_ cell had the electrode configuration 84wt% active, 8wt% PVDF binder and 8wt% Super-P carbon black; it was cycled between 3.5 and 1.8 V at C/10.

^7^Li MAS-NMR experiments were performed at 7.02 T (300 MHz) on a Bruker Avance III HD spectrometer operating at a Larmor frequency of 116.64 MHz, using a 1.3 mm MAS probe. All spectra were acquired at 67 and 60 kHz with a rotor synchronized echo pulse sequence (90°-τ-180°-τ-acq), where τ = 1/ν_r_. A π/2 pulse width of 1.6 μs was used with sufficiently long pulse recycle delays of 1 s. Spectra were collected immediately after drying (1–2 h) with 3,072 scans at a constant temperature of 283 ± 0.1 K. Chemical shifts were referenced to 1 M LiCl at 0 ppm. The spectra were normalized by the total number of scans which was the same for all runs and the weight of active materials packed in the rotors for the best possible quantitative analyses of intensity changes of each lithium resonance. Deconvolutions on the spectra were attempted in order to further gain insights into specific Li-coordination(s) via introducing least number of resonances to account for the lineshapes and asymmetries observed (best overlap > 96.5%).

## Results and discussion

**FIGURE 1:**
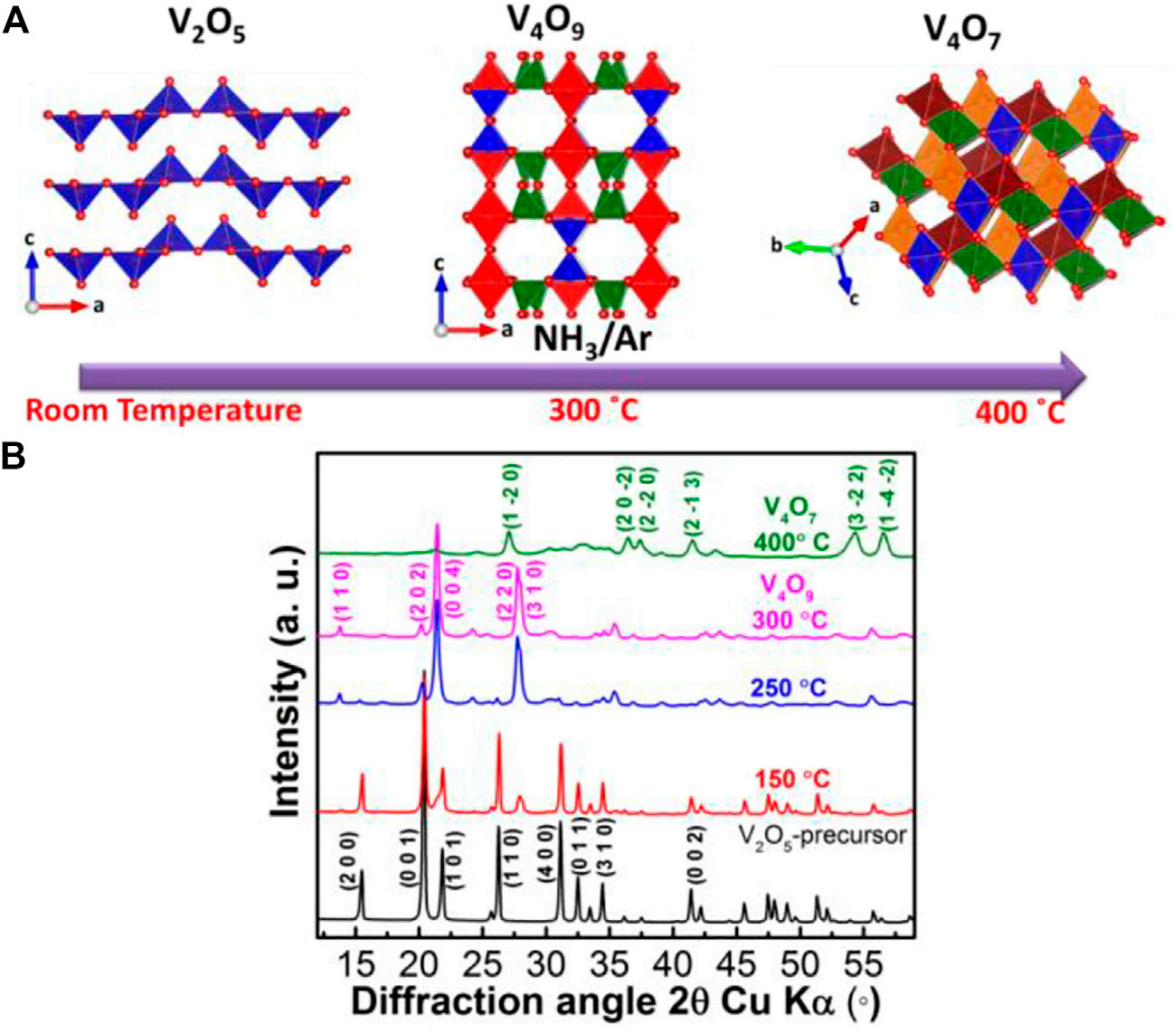
**(A)** Scheme of reduction reaction of V_2_O_5_ under the stream of 3 mol% NH_3_ in a balance of argon and **(B)** XRD patterns of vanadium oxide precursor and heat treated samples at different temperatures.

The synthesis of single phase V_4_O_9_ is complex and challenging as its formation depends on temperature as well as the strength and type of reducing agents (Marezio et al., 1973; Kawashima et al., 1975; Yamazaki et al., 2010). Improper chemical treatment leads to formation of multiple vanadium oxide phases in the sample. Herein, we found that a soft reducing agent, i.e., ammonia/argon or NH_3_/Ar gas stream can be used to synthesize single phase black color V_4_O_9_ simply from V_2_O_5_ powder precursor (Figure 1A). Figure 1B shows XRD patterns collected on vanadium oxide samples which are heat treated for 12 h at different temperatures. The pattern collected on the sample heat treated at 150°C showed the presence of additional reflections along with V_2_O_5_ precursor at 2θ = 27.4° and 28°. As the temperature was raised to 250°C, the intensity of new reflections increases at the expense of V_2_O_5_ precursor and at 300°C, only the former is visible and the corresponding new phase is identified to be V_4_O_9_. However, increasing the annealing temperature to 400°C resulted in another phase transformation from V_4_O_9_ to V_4_O_7_ (Liu et al., 2019). Thus, the product phase formed is critically tied to the reaction temperature.

Vanadium oxide V_4_O_9_, consists of an average oxidation state of + 4.5 thus possibly suggesting oxygen vacancies in the material. In fact, oxygen vacancies have been tied to better performance for oxygen deficient V_2_O_5_ (or V-V_2_O_5_); with ∼ 3% more oxygen deficiency than pristine V_2_O_5_ (based on deconvolution of XPS spectra) (Sun et al., 2018; Chotsawat et al., 2022). Certainly, at only 3% more O vacancies than pristine V_2_O_5_, the resultant global long-range structure V-V_2_O_5_ is hardly affected. The unit cell parameters of pristine V_2_O_5_ are slightly smaller than V-V_2_O_5_ due to slightly more (larger) V^4+^ ionic radii. As compared to V_4_O_9_ with theoretically 10% O vacancies (in V_2_O_5_ nomenclature), the comparisons end there. Moreover, the V-V_2_O_5_ voltage profile, just like pristine V_2_O_5_ contains steps and kinks associated with phase changes with resultant low voltage output (Sun et al., 2018), unlike V_4_O_9_ voltage profile which will be discussed later.

**FIGURE 2:**
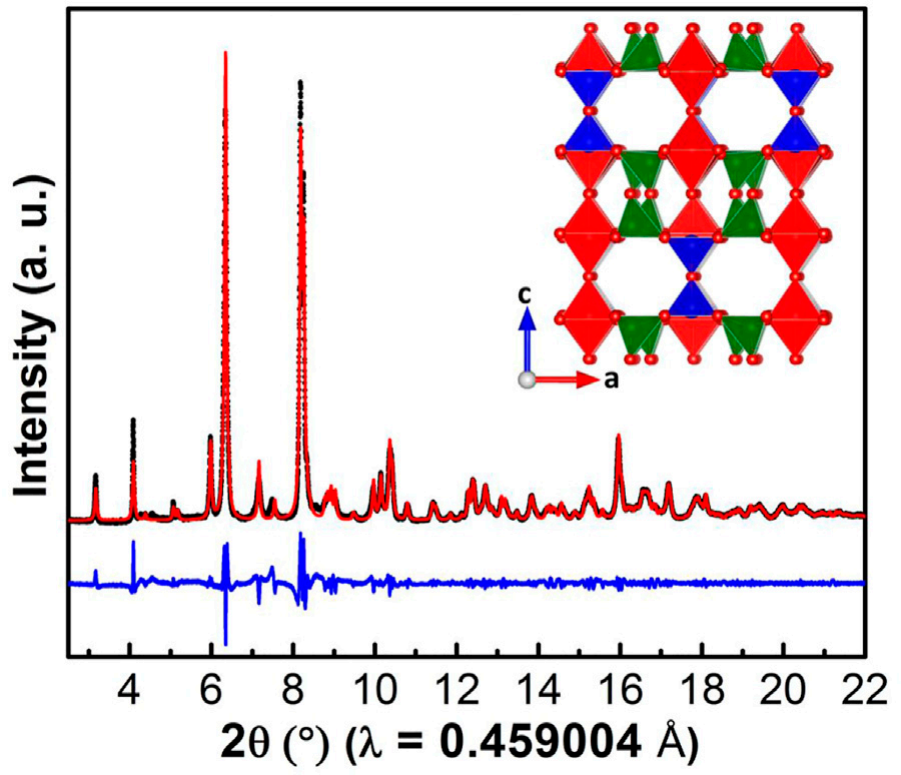
Synchrotron XRD pattern collected on V_4_O_9_ sample prepared at 300°C. The red dots and black and blue solid lines correspond to observed and calculated patterns and their difference, respectively. (Crystal structure of V_4_O_9_ is shown in inset).

Figure 2 shows the SXRD pattern of V_4_O_9_ collected using a synchrotron X-ray source. The powder pattern could be fitted well with the *Cmcm* space group where lattice parameters were found; a = 10.381 Å, b = 8.196 Å and c = 16.588 Å, in accordance to the reported values (Marezio et al., 1973; Kawashima et al., 1975; Yamazaki et al., 2010). Our V_4_O_9_ has a much larger cell volume compared to other materials made by soft chemistry routes (Wang et al., 2021), which may explain why Li ion conduction and smooth voltage profiles are observed. The crystal structure (Figure 2 inset) contains V^4+^ cations in both pyramidal VO_5_ and octahedral VO_6_ coordination, and V^5+^ cation in tetrahedral VO_4_ coordination (The Materials Project, 2022). In the ab-plane, VO_5_ square-based pyramids and VO_6_ octahedra share edges to make pairs which are then connected to VO_4_ tetrahedra through corners to form layers. These layers are further linked together via corner sharing of VO_4_-VO_4_ tetrahedra to form double layers along the *c*-axis. Further, VO_5_ pyramids and VO_6_ octahedra present in the double layers are sharing corners to make three-dimensional structure with hexagon-shaped cavities or large tunnels along the *b*-axis.

**FIGURE 3:**
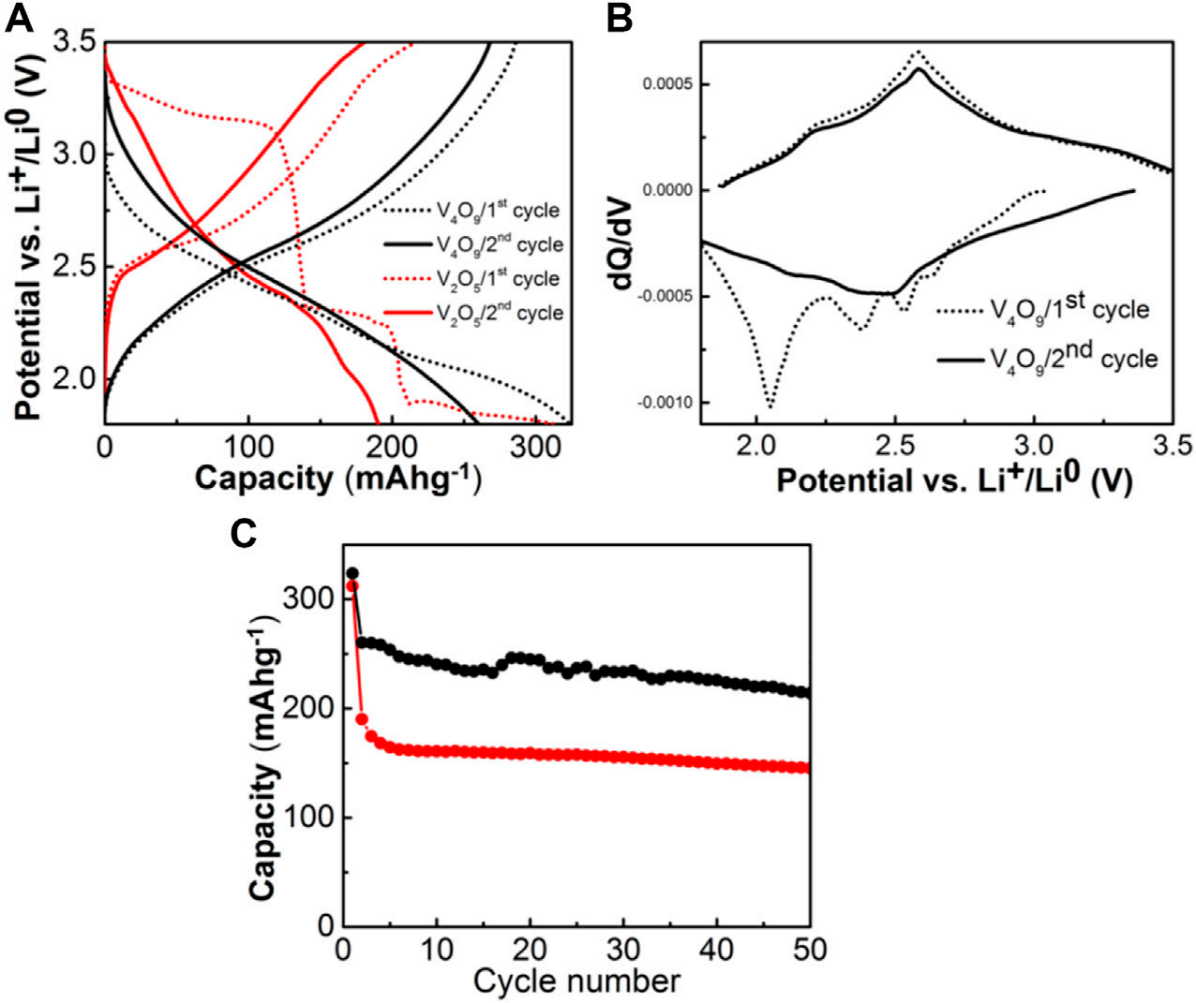
**(A)** Voltage profiles of Li/V_4_O_9_ & Li/V_2_O_5_ cells at C/10 rate in between 1.8 and 3.5 V **(B)** the dQ/dV (first two cycles) profiles of Li/V_4_O_9_ cell, and **(C)** capacity vs. cycle number plots of V_4_O_9_ and V_2_O_5_ cathodes.

For determining the V_4_O_9_ electrochemical lithium ion intercalation properties, cathodes were fabricated and cycled against Li metal at C/10 between 1.8 and 3.5 V vs. Li^+^/Li^0^. Representative data is shown in Figure 3A. During the initial discharge, the voltage gradually drops to the cutoff of 1.8 V with a capacity of 320 mAh/g. On the subsequent charge, the voltage raises smoothly back to 3.5 V vs. Li^+^/Li^0^ with a return capacity of 286 mAh/g. The coulombic efficiency cycle-to-cycle after the first cycle is above 99%. Approximately 4.2 M ratio of Li^+^ has been intercalated into the host on discharge, while only 3.7 M ratio can be extracted; these values are consistent with previous literature (Hammouche and Hammou, 1987). However, the voltage profile shapes are different as the former possesses a multitude of voltage steps whereas the material synthesized and evaluated here (i.e., “Li3.7V4O9”) exhibits, instead a classic S-shaped curve. To better explain this result, the corresponding dQ/dV curves are shown in Figure 3B which includes a set of voltage peaks or features at 2.63, 2.52, 2.38, and 2.04, and 2.21, and 2.57 V vs. Li^+^/Li^0^ during first discharge and charge respectively. These slight voltage features are eliminated or significantly muted on the second cycle.

These electrochemical results are compared to amongst the best and most recent cycling performance reported in the literature on morphologically-optimized high-performance V_2_O5 that is made in a nano-plate stacked form, whereby the capacity is stabilized at 258 mAhg^−1^ (4.0–2.0 V; 50 mAg^−1^) (Sim et al., 2023). If a nanocomposite of V_2_O_5_ made by a hydrothermal method is synthesized in the presence of conductive reduced graphene oxide (rGO) scaffold then an even higher capacity of 280 mAhg^−1^ (4.0–2.0 V; 50 mAg^−1^) was observed (Alsherari et al., 2023). Both of these recent results used nano-architecture designed materials, unlike the micron-sized V_4_O_9_ particles and their electrochemical cycling results presented here.

For the electrochemical-structural mechanism, we compare the pathway of phase transformation for the layered V_2_O_5_ prototype form versus that of V_4_O_9_ three dimensional vanadium oxide. Both phases proceed to cation disordered rock salt type structures upon different stages of lithiation. For example, V_2_O_5_ undergoes a series of phase transformations depending upon the amount of lithium inserted, α- (x < 0.01), ε- (0.35 < x < 0.7), δ- (x = 1), γ- (1 < x < 2) and ω- (x = 3) (Delmas et al., 1994). The initial transformations up to two Li per formula unit does not significantly alter the pristine structure and the intercalation occurs by puckering of VO_5_ pyramid layers. As the intercalation proceeds beyond this limit, an irreversible phase transformation results in the formation of rock salt type structure with the end composition Li_3_V_2_O_5_. This is clearly shown from the change in the voltage profile from the initial discharge to subsequent cycles as shown in Figure 3A. The puckering is too significant with deeply intercalated lithium and the vanadyl–V(V) = O bonding unit cohesiveness strength is lost. Thereafter the material still cycles, but the capacity fades somewhat significantly after 20 cycles.

**FIGURE 4:**
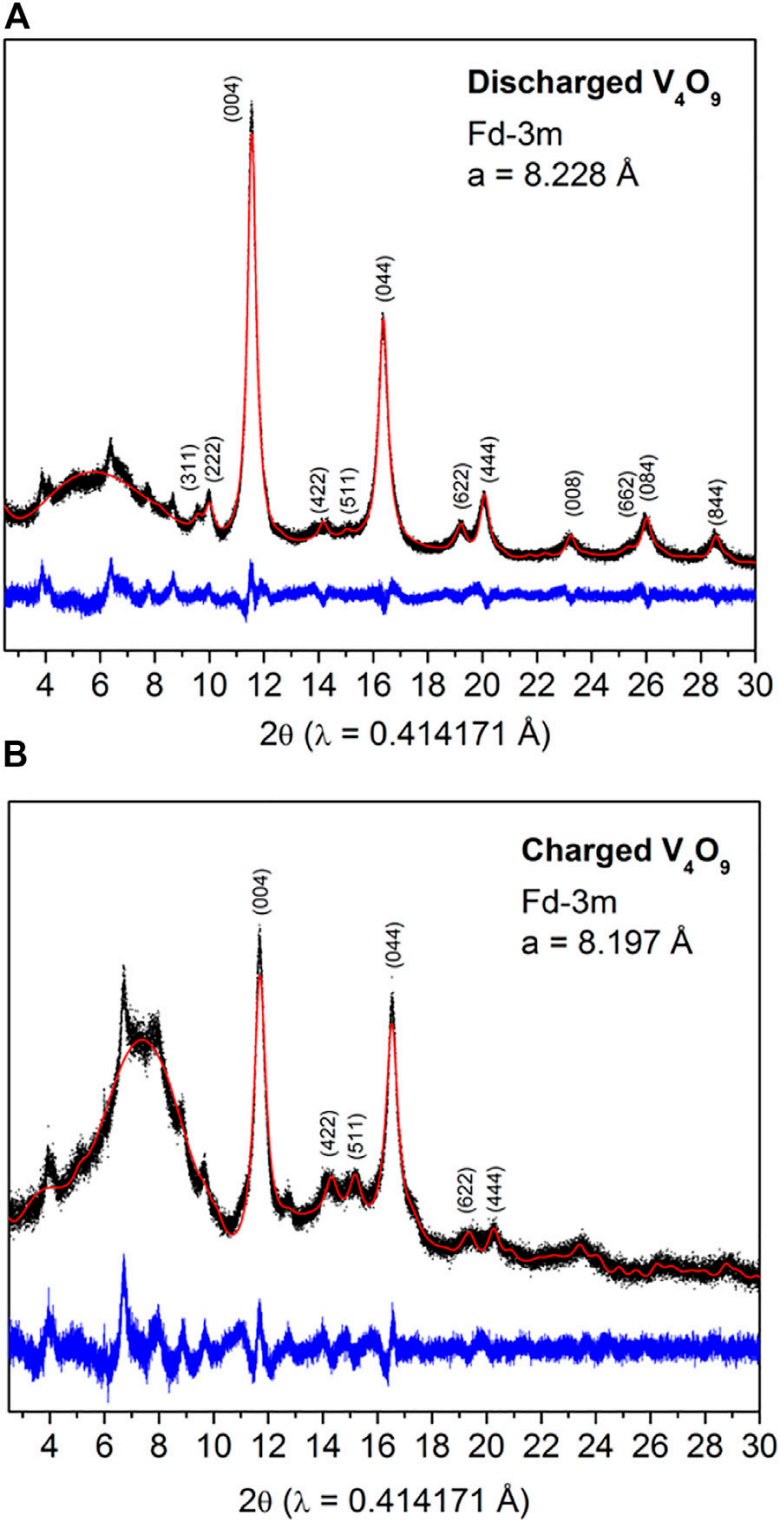
*Ex situ* synchrotron XRD patterns collected on **(A)** discharged and **(B)** charged V_4_O_9_ electrode samples. The red dots and black and blue solid lines correspond to observed and calculated patterns and their difference, respectively. The broad bump at 7° 2-theta is due to the Kapton tape that is protecting the sample from ambient air.

As demonstrated, significant changes in the voltage profiles are observed for these comparative vanadium oxide cathodes which could be attributed to successive phase transformations upon cycling (Delmas et al., 1991; Delmas et al., 1994). To compare and contrast the V_4_O_9_ results, we collected *ex situ* synchrotron XRD patterns on fully discharged and charged V_4_O_9_ electrodes during the first cycle (Figure 4). Both patterns could be partially indexed with cubic space group *Fd-3m* which is characteristic of disordered rock salt structure, as previously observed for other Li-V-O systems, such as V_2_O_5_ and LiVO_3_ (Chieh et al., 1981; Delmas et al., 1991; Pralong et al., 2012). The corresponding cell parameters of discharged and charged phase have found to be *a* = 8.228 and 8.197 Å, respectively. In addition, both patterns consist of a few un-indexed peaks which could be due to the formation of secondary phase upon electrochemical activation. These peaks do not shift or change in between discharge and charge process, thus confirming the inactivity but entrapment of some lithium as observed in the electrochemical measurements during the first cycle. Peak broadening indicates crystallographic stresses have ensued upon lithium intercalation into the host material, but does not adversely affects the electrochemical activity.

**FIGURE 5:**
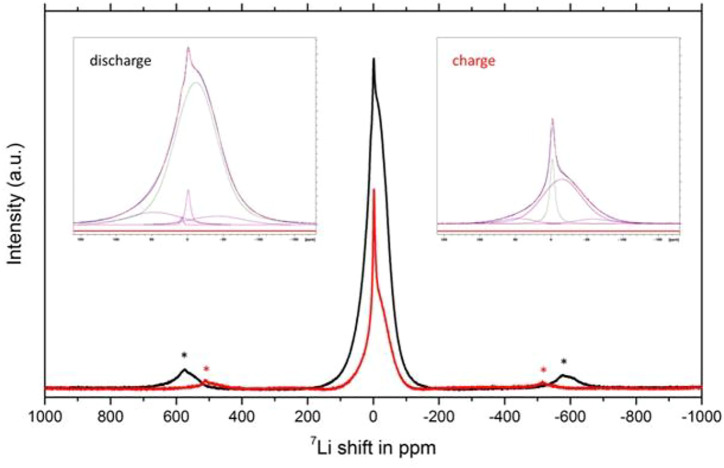
*Ex situ* ^7^Li MAS NMR spectra collected on discharged (black) and charged (red) V_4_O_9_ electrode samples. * indicates spinning sidebands. Insets show corresponding deconvolutions of the spectra.

To shed some preliminary insights into the detailed distribution of lithium ions in this disordered rock salt phase, ^7^Li MAS NMR measurements were performed on discharged and charged samples (Figure 5). Deconvolution of the spectra show a major broad resonance peak at −11.9 ppm and two minor peaks at −40 and 45 ppm, thus indicating a wide distribution of various Li cations in the structure with an envelope of associated chemical shifts. The results suggest multiple broad distributions of Li sites are available in the host lattice for lithium occupancy with respect to cycling. The intensity of these resonances diminishes to approximately 25% upon charging indicating Li removal from these sites but without disturbing the lattice. Note that the signal at 0 ppm is due to Li-containing diamagnetic species from the electrolyte/SEI.

In the electrochemical cell, V_4_O_9_ is expected to follow the similar multi-step lithium intercalation process as observed in the case of V_2_O_5_ before transforming into rock-salt type structure. We have confirmed this mechanism by *ex situ* XRD measurements. From the electrochemical results, we found that the activated rock salt phase-“Li3.7V4O9” exchanges ∼ 0.93 mol of Li^+^ per mol vanadium in the structure at an average intercalation voltage of 2.5 V vs. Li^+^/Li^0^. This is ascribed to the activity of V^5+^/V^4+^ redox couple as reported in the literature (Pralong et al., 2012). The voltage profiles of subsequent cycles exhibit S-shaped curves-a characteristic of solid-solution formation upon intercalation and de-intercalation phenomena as confirmed by our *ex situ* synchrotron XRD studies. Thanks to the low volume changes in the unit cell (ΔV/V ∼ 0.34%) and restricted redox activity confined to V^5+^/V^4+^couple (capacity value driven), the rock salt phase yields a capacity of 225 mAh/g even after 50 cycles (Figure 3C), which is higher than battery grade micron-sized V_2_O_5_.

## Conclusion

In summary, we report a simple methodology to prepare V_4_O_9_ cathode by utilizing NH_3_/Ar gas stream. The as-prepared cathode was then transformed into disordered rock salt type “Li3.7V4O9” upon electrochemical lithiation. Once it has formed, it reversibly exchanges lithium ions at an average intercalation voltage of 2.5 V vs. Li^+^/Li^0^ with an initial capacity of 260 mAh/g. With restricted redox activity V^5+^/V^4+^ and low volume changes, the disordered rock-salt type cathode renders a reversible stable capacity of 225 mAh/g after 50 cycles.

## Data Availability

The raw data supporting the conclusion of this article will be made available by the authors, without undue reservation.
